# Truth and lies in your eyes: Pupil dilation of White participants in truthful and deceptive responses to White and Black partners

**DOI:** 10.1371/journal.pone.0239512

**Published:** 2020-10-13

**Authors:** Elena Trifiletti, Stefania D’Ascenzo, Luisa Lugli, Veronica Margherita Cocco, Gian Antonio Di Bernardo, Cristina Iani, Sandro Rubichi, Roberto Nicoletti, Loris Vezzali

**Affiliations:** 1 Dipartimento di Scienze Umane, Università di Verona, Verona, Italy; 2 Dipartimento di Filosofia e Comunicazione, Università di Bologna, Bologna, Italy; 3 Dipartimento di Educazione e Scienze Umane, Università di Modena e Reggio Emilia, Reggio Emilia, Italy; 4 Dipartimento Chirurgico, Medico, Odontoiatrico e di Scienze Morfologiche con Interesse Trapiantologico, Oncologico e di Medicina Rigenerativa, Università di Modena e Reggio Emilia, Reggio Emilia, Italy; 5 Dipartimento di Scienze Biomediche, Metaboliche e Neuroscienze, Università di Modena e Reggio Emilia, Reggio Emilia, Italy; 6 Centro Interdipartimentale di Neuroscienze e Neurotecnologie, Università di Modena and Reggio Emilia, Italy; University of Connecticut, UNITED STATES

## Abstract

In the present study, we examined the pupillary response of White participants who were asked to tell the truth or lie to White or Black partners. Research on cues to deception has assumed that lying is more cognitively demanding that truth telling. In line with this assumption, previous studies have shown that lying is associated with greater pupil dilation, a behavioral cue that typically manifests itself under conditions of stress or cognitive effort. In accordance with these results, we predicted greater pupil dilation when lying than when telling the truth. Furthermore, pupil dilation was expected to be greater when responding to White than Black partners. Finally, we hypothesized that pupil dilation would be greater when lying to White than Black partners. Participants were instructed to answer a set of questions, half truthfully and half deceptively. They were led to believe that White vs. Black partners (one male and one female) would ask the questions via computer connection. Indeed, we used feminine and masculine synthetic voices. Pupil dilation was assessed with a remote eye-tracking system. Results provided support for the first two hypotheses. However, the predicted interaction between race of partners and truth status of message (lying vs. telling the truth) was nonsignificant. Our findings highlight the importance of considering race in the study of truthful and deceptive communications.

## Introduction

Lying is a significant part of our daily social life [e.g., 1]. One of the first studies about the role of lies in everyday life found that people tell on average one or two lies each day [2]. Lying involves the whole spectrum of human social interactions, from intimate relationships [3–5] to job interviews and workplace relationships [6, 7] to forensic settings [8]. Unsurprisingly, lying and deception have been a topic of prominent interest for centuries. Empirical research has focused on the two related topics of accuracy of deception judgments and behavioral cues to deception [9, 10]. In particular, the question of whether people behave in discernibly different ways when they are lying compared to when they are telling the truth has interested academics, practitioners, and laypeople for a long time [11, 12].

Traditionally, research on cues to deception has examined individuals’ verbal and nonverbal behavior by means of human observation or technological equipment assessing physiological and neurological responses, such as the polygraph, voice-stress analyzers, electroencephalograms, and, more recently, eye-tracking systems and functional magnetic resonance imaging (fMRI). Scientific meta-analyses and reviews on cues to deception provide support for the idea that lying is accompanied by specific physiological and neurological alterations, involving for example pitch [10, 13], pupil dilation [10], activation of prefrontal cortex, anterior cingulate cortex, and parietal cortex [14, 15].

In the present study, we used pupil dilation as a cue to deceptive messages. Importantly, we considered for the first time the role of group membership, by assessing changes in pupillary responses of White participants when lying or telling the truth to White or Black (fictious) interaction partners. Despite overwhelming evidence that race plays a key role in regulating cognition, affect, and behavior, this factor has been surprisingly overlooked in cues to deception studies. Whereas recent lie detection research has examined receivers’ accuracy when judging the truthfulness of own-race and other race liars and truth tellers [16; see also 17, supplemental materials], scarce attention has been paid to how race of receivers affects the behavior of liars and truth tellers. In the present research, we argued that White participants’ affective and cognitive processes involved in deceptive and truthful responses directed at White and Black partners may diverge, thus activating different physiological or behavioral responses.

### Pupil dilation and lie detection

Changes in the diameter of the pupil typically reflect an autonomic and uncontrollable reaction [but see 18] that is activated in response to fluctuations in ambient light, as well as to on-going cognitive effort, interest, surprise, uncertainty, or other emotions [19–23; for reviews see 24, 25]. Pupil dilation can result from sympathetic nervous system stimulation or suppression of the parasympathetic nervous system, under conditions of stress, cognitive effort, arousal, and pain [e.g., 26–30; for reviews see 31]. Since it is typically unconscious, the pupillary response authentically reflects people’s inner states [29], and thus it may be a particularly useful source of information for identifying the veracity of another person’s statements.

Studies in the lie detection literature have shown that pupil dilation may be a valid cue to deception. Lying may be stressful or cognitively difficult and this has an impact on pupil dilation [32]. For instance, Berrien and Huntington [33] found greater pupillary response among participants attempting deceit than those not attempting deceit, when critical questions were first introduced. Heilveil [34] showed that when participants declared to respond deceptively their pupils were dilated significantly more than when they declared to respond truthfully. In a study by Dionisio, Granholm, Hillix, and Perrine [35], participants were asked to answer the same set of questions twice, once truthfully and once deceptively, while their pupil size was recorded. Pupil dilation was significantly greater for deceptive than for truthful answers. Wang et al. [32] found that pupil size increased before and after communicating a deceptive message in a strategic game, and this increase was positively correlated with the magnitude of deception. Hochman, Glöckner, Fiedler, and Shavar [36] further confirmed this result, showing that pupil dilation is greater when participants are cheating to maximize their payoff in a dots task. Additional evidence was provided by Lubow and Fein [37], who used a concealed recognition paradigm to examine the effects of committing a mock crime on pupil dilation. After being assigned to the “innocent” or “guilty” group, participants completed a visual guilty knowledge test, wherein pictures of relevant (target) and irrelevant (control) details of the mock crime scene were presented. Results showed that target items elicited grater pupillary response than control items for “guilty” but not for “innocent” participants (Experiment 2). Overall, these findings suggest that pupil dilation is a valid cue to deceptive behaviors.

### Intergroup influences and pupil dilation

Human cognition heavily relies on categorization of physical and social stimuli to provide meaning, order, and structure to our knowledge [38]. Race, as well as the other basic demographic distinctions of age, gender and social class, serves as a chronically salient social category [39, 40] that is processed instantaneously. Research recording electrocortical responses has shown that the amplitude of P200 (which is associated with early attention) is typically larger for White participants exposed to pictures of Black than White targets [41, 42], while this pattern is reversed for Black participants [43], thus suggesting that other-race targets activate early attention responses [44]. Racial categorization elicits relevant stereotypes and affective responses, which are reflected in a wide array of neural and physiological correlates [45]. For instance, past research employing physiological measures found that White participants showed larger electrodermal responses [suggesting greater anxiety; 46] and cardiovascular activity denoting threat [47] when interacting with a Black rather than with a White experimenter. These reactions are likely the result of negative stereotypes and prejudice feelings activated by race categorization. Indeed, numerous studies have found greater activation of the amygdala in response to outgroup than ingroup face stimuli [48, 49].

At the same time, research has shown that human beings exhibit greater concern for the welfare of fellow ingroup members [50]. The fact that shared social identity triggers a preference for the ingroup over the outgroup is well-documented in a diverse array of studies. For instance, research on the cross-race effect has consistently shown that own-race faces are better recognized compared to cross-race faces [51]; this effect is associated with greater activity in the fusiform region (a brain area linked to encoding of faces) for own-race than cross-race targets [52]. A number of studies using the dot-probe detection paradigm [53–55] also showed that, albeit attending more to Black than White faces in the first stages of visual attention, White participants pay more attention to own-race over cross-race faces in later stages. This result is further confirmed by electrocortical research showing larger amplitude of N200 for own-race than cross-race faces [42, 43]. Other studies suggest that unconscious emotional and behavioral mimicry is greater for ingroup than outgroup members [e.g., 56, 57; but see 58].

Evidence of ingroup preference in social interactions also comes from studies employing physiological markers, in particular eye gaze and pupil dilation. A variety of studies have shown that White and Asian participants make more fixations and attend more to the eyes of ingroup than outgroup faces [59–61]. Kret et al. [29] examined participants’ pupil dilation in response to pupillary changes of a White versus Asian interaction partner. Results showed that participants’ pupils were larger, dilated faster, and showed a greater peak when observing a partner with dilating pupils compared to a partner with static pupils, and this mimicry was stronger for own-race than other-race partners. Another example is provided by Azevedo et al. [62] who combined event-related fMRI with measurements of pupil dilation. Pupillary response and activation of left anterior insular cortex of both White and Black participants were greater when they were exposed to own-race than to other-race pain. In sum, these results suggest that human beings are especially motivated to pay attention to and preferentially maintain social bonds with fellow ingroup members. Based on these considerations, in contexts where one's credibility is judged by others, cognitive effort should be greater when these others are members of one’s own group rather than members of an outgroup.

Another intergroup influence that may affect how participants react when asked to tell the truth or lie in an intergroup context is status. Social interactions are deeply shaped by social status, and human beings are particularly sensitive to social hierarchies [63]. When examining interracial relations, it is important to note that racial categories are assigned different levels of status and power in society. Greater prestige and power is usually accorded to Whites than Blacks. Since those who have higher status have more resources to punish individuals that are identified as deceivers [see 64], compared to those with lower status, participants might experience more pressure when their truthfulness is judged by White (high-status) than when it is judged Black counterparts (low-status). In addition, research has found that attentional resources are preferentially allocated to high-status individuals [e.g., 63, 65].

Overall, both the argument of greater concern for the ingroup and the argument of sensitivity to social status suggest that greater cognitive effort (hence, greater pupil dilation) should be involved when White participants’ truthfulness is judged by White than by Black counterparts. On the contrary, the activation of racial stereotypes and prejudice should activate a more intense emotional reaction in the case of a Black counterpart. However, the explanation based on racial prejudice seems less likely to play a role in a context where one’s credibility is in the spotlight. In such a context, individuals should be concerned about how they will be judged by fellow ingroup members (concern for the ingroup) and/or motivated to avoid social sanction (sensitivity to social status).

### The present research

In this study, we examined the pupillary response of White participants who were asked to tell the truth or lie to White or Black interaction partners. Previous research has examined autonomic changes in pupil size of participants who voluntarily provided a truthful or a deceitful message or that were induced to do so. These studies showed that pupil size increases for deceptive behaviors [e.g., 32, 35], possibly because of greater cognitive difficulty and emotional burden associated with lying. However, research has not yet examined whether and how pupil size changes depending on the race of the target of truthful versus deceptive messages. Based on the above reported arguments about preference for maintaining social bonds with ingroup members and sensitivity to social status, we reasoned that being judged by a White counterpart should be more stressful for White participants than being judged by a Black counterpart.

To test this idea, we designed an experiment in which White participants were asked to answer a set of questions, half deceptively and half truthfully. They were led to believe that two other participants (one female and one male)–both White or Black, depending on the experimental condition–would ask the questions through a computer audio connection from an adjacent room. Pupillary responses of participants were recorded with eye tracking technology while answering the questions.

In line with previous research on cues to deception, we predicted greater pupillary response for deceitful than truthful answers (*Hypothesis 1*). Moreover, drawing on the arguments of greater concern for the ingroup and sensitivity to social status, greater pupil dilation was expected when White participants were responding to White than Black partners (*Hypothesis 2*) as a signal of greater attention or cognitive effort. Lastly, we predicted that truth status (telling the truth vs. lying) would interact with race of the partner, so that pupil size would be greatest when lying to White vs. Black partners (*Hypothesis 3*), because the cognitive effort required by lying adds up to that of interacting with a White partner.

The research was carried out in Italy, where racial conflict has become a relevant issue more recently than in other Western countries. In Italy, as well as in other Western Europe countries, the number of immigrants has substantially increased in the last decades and this has led to renewed manifestations of prejudice and nationalist sentiments [see 66, 67], therefore making the categorization based on race as especially meaningful.

## Materials and methods

The study was approved by the Ethics Committee of the Department of Human Sciences at the University of Verona (Italy). The approval number is 2017_25. Participants provided written informed consent prior to their participation.

### Participants

Participants were 44 White undergraduate students recruited at the University of [OMITTED FOR BLIND REVIEW] who took part in the experiment on a voluntary basis. Four participants were discarded from the analyses (see Data preparation), thus leaving a sample of 40 participants (20 females, 20 males; mean age = 20.63 years, *SD* = 3.48). All participants self-identified as White. Participants were randomly assigned to one of two experimental conditions: White versus Black interaction partners. The study was approved by the Ethics Committee of the Department of [OMITTED FOR BLIND REVIEW] at the University of [OMITTED FOR BLIND REVIEW]. All participants provided written informed consent to participation in the study and to the inclusion of data pertaining to themselves.

### Apparatus, stimuli and procedure

The experiment was conducted in a quiet room, where the light was dimmed. Participants’ eye movements were monitored with the eye tracker system SMI 500 by SensoMotoric Instruments (http://www.smivision.com) at 250 Hz sampling rate. Stimuli were presented on a Dell 22-inch (56 cm) video monitor (refresh rate: 60 Hz; resolution: 1680 x 1050 pixels). The viewing distance was 60 cm. Stimuli presentation and data acquisition were controlled by E-Prime Professional v2.0 software (http://www.pstnet.com).

Audio stimuli (questions) were obtained with a vocal syntethizer. Originally, we generated a female and a male voice (www.oddcast.com) in order to match sex of the synthetic voice with sex of participants. However, the duration of the audio stimuli ranged between 1042 ms and 2278 ms for the female voice, and between 834 ms and 2000 ms for the male voice. Because of this difference, each participant was presented with both types of voices, in order to control for possible effects of unbalanced stimuli duration. Please note that one female voice was adopted for both female partners (White/Black) and one male voice was adopted for both male partners (White/Black).

Upon arrival at the laboratory, participants were seated in front of the computer screen. They were told that two other (fictious) participants (one female and one male) were placed in an adjacent room, and that interaction with them would take place via computer connection. Participants were further explained that the two partners would ask, one after the other, a list of yes-no questions, and that, due to methodological requirements, they would write each question on a keyboard; the questions would be in turn transformed through a vocal syntethizer. The task of participants was to answer each question either truthfully or deceptively, according to the instructions provided on the computer screen immediately before each question. Participants were told that each of the two partners would ask them the same list of questions. In order to increase the relevance of the task, they were also informed that each partner would judge their truthfulness. With the aim of increasing the credibility of the manipulation, prior to answering the questions, participants were shown two video-clips in which each of the two partners presented him/herself. Half of the participants watched video-clips of two White confederates (a male and a female) and the other half video-clips of two Black confederates (a male and a female). All participants identified the White confederates as White and the Black confederates as Black, respectively. Printed pictures of the two confederates were positioned on the desk, next to the computer screen, and remained visible for the entire duration of the alleged interaction. To further enhance the credibility of the interaction, participants were invited to record their own video-clip and to take a picture of themselves; they were led to believe that these materials would be shown to the two partners.

The list of questions included six practice questions and 36 test questions (the full list is provided in Table 1), which were presented auditorily (by means of synthetic voices). Examples of audio stimuli used in this study are available in Open Science Framework at. https://osf.io/r4gha/?view_only=710c875a0ecd4d73b47c19b6a2b63f98 [doi: 10.17605/osf.io/r4gha]. For each partner, half questions had to be answered truthfully and the other half deceptively; the instruction for each question was the same across the two fictitious partners and it was presented in a pseudorandom order (identical across participants), with truthful and deceitful instructions intermixed. In order to check whether participants had answered in accordance with the instructions, at the end of the experimental session they were asked to answer the same questions again; the questions were written on a sheet of paper. Participants were instructed to answer truthfully to each question. In order to minimize possible effects of categorization based on sex, order of partners was counterbalanced, so that half participants were randomly assigned first to the same-sex partner and the other half were assigned first to the other-sex partner.

**Table 1:** List of practice and test auditory questions.

Question	Type of trial
Are you a sportsman/woman?	Practice
Do you have a dog?	Practice
Do you have a cat?	Practice
Do you have a smartphone?	Practice
Are you over 20?	Practice
Do you have four grandparents?	Practice
Is your zodiac sign Aquarium?	Test
Does your name end with the letter A?	Test
Do you like the mountains?	Test
Do you play tennis?	Test
Do you like going to the cinema?	Test
Do you have a tattoo?	Test
Are you from Bologna?	Test
Do you live with your family?	Test
Do you like going to the disco?	Test
Do you go to university by bike?	Test
Do you have a blue car?	Test
Do you like skiing?	Test
Have you ever travelled by plane?	Test
Have you ever been in America?	Test
Do you like cooking?	Test
Do you like reading?	Test
Do you go to the gym?	Test
Do you attend a yoga class?	Test
Are you on Facebook?	Test
Are you engaged?	Test
Do you have any brothers or sisters?	Test
Your father’s name is Giacomo?	Test
Are you a student worker?	Test
Do you have a passion for photography?	Test
Do you have a scooter?	Test
Do you like horror movies?	Test
Do you play an instrument?	Test
Do you like rock music?	Test
Do you like drinking wine?	Test
Were you born in April?	Test
Have you ever run a marathon?	Test
Have you ever failed an exam?	Test
Do you have a driver’s license?	Test
Is your family large?	Test
Do you like the countryside?	Test
Have you ever attended a language course?	Test

**Fig 1:**
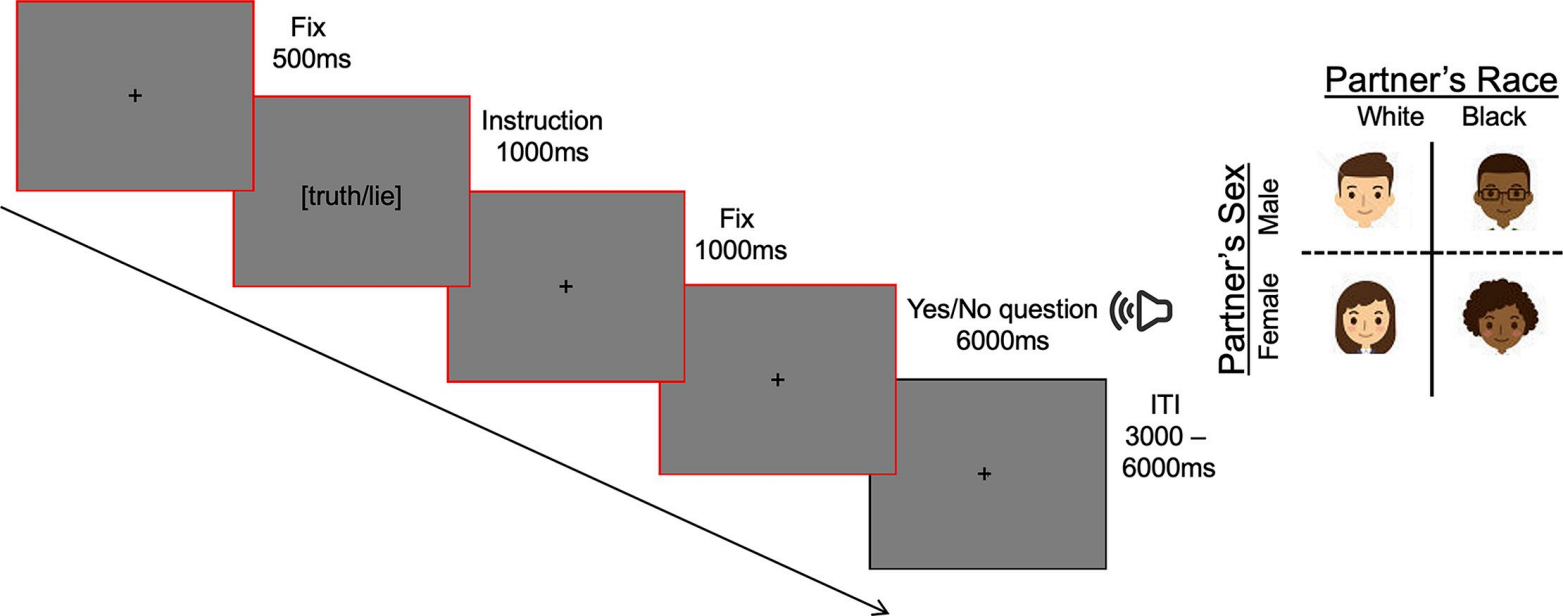
Leftmost panel. Temporal sequence of a representative trial. A red square indicates when the pupillary response was recorded. Partner’ s sex was varied within participants, while the Partners’ race was a between-participants factor.

Each trial started with a fixation cross for 500 ms, followed by the truth vs. lie instruction for 1,000 ms and a second fixation cross for 1,000 ms. During the following 6,000 ms the question was presented through two loudspeakers, placed 15 cm on the left and on the right side of the computer screen, respectively. Participants pressed a key on the left or on the right of a QWERTY keyboard (“z” and “m” keys, respectively) to answer “yes” or “no” in accordance with the instruction received for that question (see Fig 1). The intertrial interval randomly ranged between 3,000 and 6,000 ms.

### Data preparation

Pupil size was acquired continuously, at a rate of 250 samples per second, from the right eye. Data were converted and analyzed using ILAB [68] and in-house developed MATLAB routines [see 69]. Pupil diameters with missing values or values above or below 2.5 standard deviations from the average pupil diameter in each trial were excluded from data analysis. Trials in which large pupil changes (> 0.3 mm) occurred or with an exceedingly small pupil size (< 1 mm) were excluded. Similarly, data segments of less than 5 samples, which were preceded and followed by excluded data, were discarded from data analyses. If a trial contained more than 50% blinks or excluded data, it was entirely discarded from data analyses (for the same procedure see also 27; 69]. Linear interpolation was used to replace excluded data (starting from 5 samples before and ending 5 samples after) in the remaining trials. Trials with inaccurate answers (not matching the truth-lie instruction) were not included in the analyses.

The baseline pupil size was defined as the average pupil size (mm) of the 500 ms interval at the beginning of each trial (fixation cross). In each trial, the baseline pupil size was subtracted from the pupil diameter changes following stimulus onset and was subtracted from the time series of the tracking period. Trials within each cell of the experimental design (i.e., Truth status of the message; Partners’ race) were averaged for each participant. Moreover, data were collapsed across the female and male audio stimuli. For statistical analyses, these pupil diameter changes were then averaged between 4,000 ms and 8,500 ms based on pupil dilation observation. Response latencies (from the onset of each audio stimulus, i.e. the question) were also collected, even though this was not a focal dependent variable in the present study.

Three participants were excluded from the analyses because more than 50% of their total trials were discarded due to signal loss and one participant was excluded because failing to comply with the instructions (for more than 20% of the total trials). As a result, as reported in the Participants section, 40 out of 44 participants were included in the analyses.

## Results

The data that support the findings of this study are openly available in Open Science Framework at https://osf.io/r4gha/?view_only=710c875a0ecd4d73b47c19b6a2b63f98 *[doi*: *10*.*17605/osf*.*io/r4gha]*

Preliminary analyses revealed that the order with which participants were assigned to same-sex or other-sex partners (same-sex vs. other-sex first) and sex of participants did not affect pupil size results. Therefore, these factors were excluded from analyses. A 2 (Partners’ race: White vs. Black) × 2 (Truth status: truth vs. lie) mixed ANOVA with the last factor as a within-participants factor was applied to pupil size data. The 2 × 2 ANOVA was subsequently applied to response latencies for exploratory purposes. We computed a post-hoc sensitivity power analysis using GPower [70], with 80% of power, alpha = .05, and correlation between repeated measures = .66. The analysis yielded an effect size f of 0.39 (corresponding to η^2^_p_ = .134) for the main effect of Partners’ race, an effect size f of 0.22 (corresponding to η^2^_p_ = .047) and of 0.16 (corresponding to η^2^_p_ = .025) for the main effect of Truth status and its interaction with Partners’ race, respectively.

**Fig 2:**
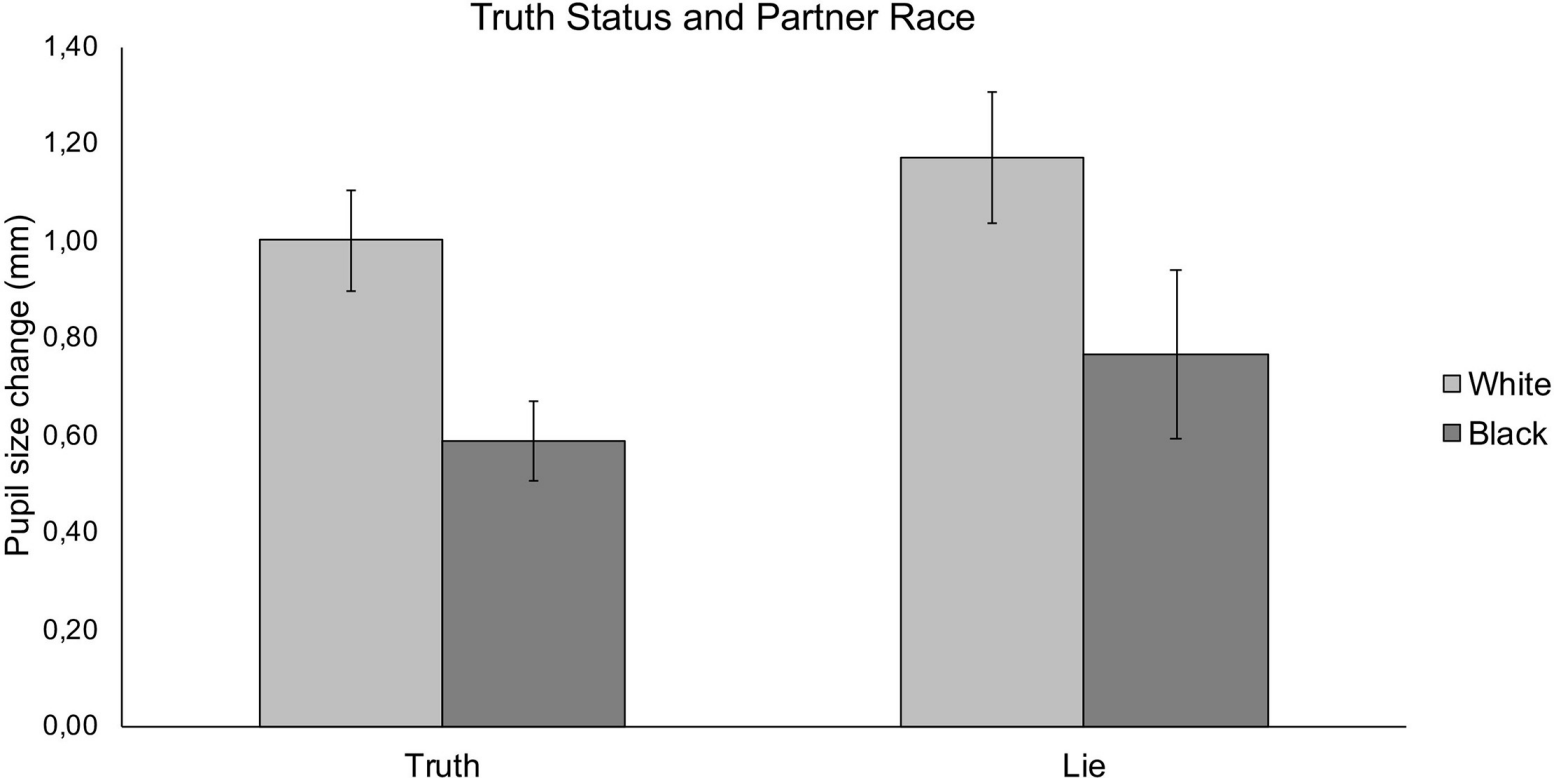
Mean pupil size change (mm) as a function of truth status and partners’ race. Error bars indicate standard errors of the mean adjusted for within participants design [71].

When considering pupil size data, a main effect of Truth status emerged, *F*(1,38) = 4.20, *p* = .047, η_p_^2^ = .10. In line with *Hypothesis 1*, participants displayed greater pupil dilation for deceitful (*M* = 0.97, *SD* = 0.71) than truthful messages (*M* = 0.79, *SD* = 0.47, see Fig 2, leftmost panel). We also obtained a significant main effect of Partners’ race, *F*(1,36) = 6.49, *p* = .02, η_p_^2^ = .15. Supporting *Hypothesis 2*, results revealed greater pupil dilation when participants answered the questions of White (*M* = 1.09, *SD* = 0.51) than Black partners (*M* = 0.68, *SD* = 0.51, see Fig 2, rightmost panel). The expected interaction between Partners’ race and Truth status (*Hypothesis 3*) was nonsignificant, *F*(1,38) = 0.003, *p* = .96, η_p_^2^ = .00. Overall, findings supported *Hypotheses 1* and *2*, whereas they did not support *Hypothesis 3*. An additional ANOVA was performed on pupil size data in which Partner’ s sex (female vs. male) was included as an additional factor to control for its effects. Results replicated those reported in the previous ANOVA and showed that neither the main effect of Partner’ s sex (*p* = .436) nor its interaction with the other factors (*F*_*s*_ <1) were significant.

Finally, results concerning response latencies revealed a significant main effect of Truth status, *F*(1,38) = 53.13, *p* < .001, η_p_^2^ = .58, with slower responses for deceitful (*M* = 2,555.88, *SD* = 387.90) than truthful answers (*M* = 2,300.61, *SD* = 302.44). The main effect of Partners’ race, *F*(1,38) = 0.68, *p* < .42, η_p_^2^ = .02, and its interaction with Truth status, *F*(1,38) = 0.20, *p* = .66, η_p_^2^ = .01, were nonsignificant.

## Discussion

In the present study, we tested pupil dilation of White participants as a cue to deception in interactions with White and Black counterparts. The results highlight the importance of merging research on cues to deception and race processing. Specifically, our findings extend past empirical work by showing that pupil dilation of White participants is greater when responding to White than Black partners. This result is consistent with the claim that cognitive effort is greater when the truthfulness of one’s statements is judged by ingroup rather than outgroup members and that individuals are especially motivated to maintain social bonds with fellow ingroup members. This is in line with results showing that individuals care more about their reputation with ingroup than outgroup members [72]. Another possible explanation is that differences in the pupillary response to White and Black partners depend on the status of the ethnic group examined. Whites are usually accorded higher status (and power) in society. One may argue that the situation in which one’s truthfulness is judged by high-status individuals (e.g., Whites) is more stressful compared to the situation in which one’s credibility is judged by low-status individuals (e.g., Blacks), because individuals with high-status in society have more resources to punish those that are identified as deceivers [see 64], compared to individuals with a low status. If so, then the pupillary response of Black, or of participants belonging to other minority groups, should follow the same pattern shown by White participants in the present study. Although the possible underlying mechanisms of pupillary changes in White and Black partner conditions were not investigated in this study, the observed results suggest that race is a factor that deserves attention also in the field of truthful and deceptive communications.

Contrary to our expectations, pupil dilation was larger in interactions with White than Black partners regardless of whether White participants were telling the truth or lying. Although we expected that pupil dilation would be larger when lying to White than to Black partners, our findings suggest that this is not the case. One possible explanation for this result is that participants might have anticipated greater costs for being judged as liars by White than by Black counterparts, and this might have engendered greater concerns of appearing truthful when answering to White partners, independently from the truthfulness or deceptiveness of the answer. This interpretation is in line with research on the black sheep effect, which has shown that people differentiate to a greater extent between normative and deviant ingroup members than between normative and deviant outgroup members [73], and that individuals hold expectations about this differential evaluation from a young age [74].

In interpreting these findings, it is important to consider that participants did not spontaneously choose whether or not to lie. Rather, they answered deceptively or truthfully according to the instructions provided (for differences underlying spontaneous vs. instructed lying and truth-telling, see, e.g., [75]). This might have caused similar levels of distress associated with deceptive and truthful answers. Future research should verify whether spontaneous truthful versus deceitful accounts elicit different pupillary responses.

The result that race affects pupil dilation as a cue to deception may have implications for the development of more efficient lie detection machines, especially technologies that use behavioral cues associated with deception. For instance, Gonzalez-Billandon et al. [76] have recently developed a machine learning system to detect lies that is based on several behavioral cues, including pupil dilation. Our findings suggest that race should be considered in the development of such technologies and that its effects should be further tested considering other relevant behavioral cues to deception.

It is worth noting some limitations of the present research. First, the underlying mechanisms of pupil dilation were not tested. As previously noted, there are at least two possible alternative explanations of the effect observed in this study, namely concern of appearing truthful and the relative social status of the ethnic groups involved. Future research should investigate if these factors mediate the effects of race on pupil dilation. Second, the present study only investigated White participants’ responses to (fictitious) White or Black interaction partners. As already mentioned, there might be differences between ethnic majority and minority group members’ responses in intergroup settings; moreover, participants’ reactions in truthful and deceptive interactions might depend on the specific intergroup relationship examined. Future studies should test the pupillary response of both minority and majority group members and consider a variety of intergroup relationships. Third, we did not test the role of moderating factors, such as the severity of consequences if lies are detected. Research on lie detection has suggested that high-stakes lies are generally more difficult to tell than low-stakes lies [77], in part because emotions such as fear or remorse [78] must be convincingly conceived. This likely manifests in salient behavioural signs or ‘leakage’ [79]. It is therefore possible that under high stakes conditions a greater pupillary response would be observed when lying rather than when telling the truth. Fourth, studies in the lie-detection literature usually use tasks where participants are incentivized to lie. In the present study, however, we instructed participants to lie or tell the truth without providing any incentive. Nonetheless, our results are in line with previous studies showing that pupil dilation is significantly greater when lying than when telling the truth. Finally, the use of synthetic voices could have made the participants perceive the experimental setting as somewhat artificial. Although modern speech synthesis systems simulate real human voices quite well, they still have some limitations, in terms of naturalness, prosody, and spontaneous speech [80].

Notwithstanding these limitations, the present paper provides a novel contribution to the literature, by merging research on cues to deception with research on intergroup interactions. Our results significantly expand prior research by demonstrating that White participants’ pupils are more dilated when interacting with White than with Black individuals. Findings of the present study also provide support to the results of previous research on physiological cues to deception, showing larger pupil dilation when lying than when telling the truth. In conclusion, we believe that the present study suggests a promising avenue for future research by showing that race is a relevant factor to investigate in truthful and deceptive communications.
