# Correction: Truth and lies in your eyes: Pupil dilation of White participants in truthful and deceptive responses to White and Black partners

**DOI:** 10.1371/journal.pone.0244009

**Published:** 2020-12-09

**Authors:** Elena Trifiletti, Stefania D’Ascenzo, Luisa Lugli, Veronica Margherita Cocco, Gian Antonio Di Bernardo, Cristina Iani, Sandro Rubichi, Roberto Nicoletti, Loris Vezzali

The second affiliation for the sixth author is incorrect. Cristina Iani is not affiliated with #5, but with #6: Centro Interdipartimentale di Neuroscienze e Neurotecnologie, Università di Modena and Reggio Emilia, Italy
